# Pearls and perils of an implantable defibrillator trial using a common control: implications for the design of future studies

**DOI:** 10.1186/1745-6215-9-24

**Published:** 2008-05-02

**Authors:** Peter J Kudenchuk, Alfred P Hallstrom

**Affiliations:** 1 The Department of Medicine, Division of Cardiology, and the Department of Biostatistics, University of Washington, Seattle, WA, USA

## Abstract

**Aims:**

Implantable defibrillators are considered life-saving therapy in heart failure (CHF) patients. Surprisingly, the recent Sudden Cardiac Death in Heart Failure Trial (SCD-HeFT) reached an opposing conclusion from that of numerous other trials about their survival benefit in patients with advanced CHF. A critical analysis of common control trial design may explain this paradoxical finding, with important implications for future studies.

**Methods and Results:**

Common control trials compare several intervention groups to a single rather than separate control groups. Though potentially requiring fewer patients than trials using separate controls, variation in the common control group will influence all comparisons and creates correlations between findings. During subgroup analyses, this dependency of outcomes may increase belief in the presence of a real subgroup effect when, in fact, it should increase skepticism. For example, a high (r = 0.92), statistically unlikely (p = 0.052) correlation between comparisons was observed across the subgroups reported in SCD-HeFT. Such concordance between amiodarone and a defibrillator across subgroups was unexpected, given how much the effects of these treatments significantly differed from one another in the main study. This suggests the study's subgroup findings (specifically the absence of benefit from defibrillators in advanced CHF) were not necessarily a consequence of treatment; more likely, they resulted from variation in what the treatments were compared against, the common control.

**Conclusion:**

Common control trials can be more efficient than other designs, but induce dependence between treatment comparisons and require cautious interpretation.

## Background

Implantation of a cardioverter defibrillator is considered life-saving therapy in patients with moderate to severe symptoms of congestive heart failure (CHF) and diminished ventricular function. In most defibrillator trials, patients with "sicker" hearts derived the largest survival benefit from such treatment [1-4]. Surprisingly, a recent randomized trial, Sudden Cardiac Death in Heart Failure (SCD-HeFT), reached an opposing conclusion [5]. The so-called "SCD-HeFT paradox" was the puzzling finding that survival in the subgroup of patients with New York Heart Association (NYHA) Class III CHF was not improved by receipt of an implantable defibrillator. This apparent contradiction may not, however, represent a true clinical phenomenon, but rather a consequence of trial design.

SCD-HeFT was somewhat unusual among defibrillator trials in having used a common control design. Our purpose here is to describe the statistical principles that are foundational to trials using a common control and how such a design may provide insight into apparent paradoxical outcomes in subgroup analyses. The rising popularity of such trials requires that clinicians have a working knowledge of these principles. For statistically oriented readers, an appendix (see additional file 1) provides some derivations and formulae.

## Methods

### Common control versus parallel trial designs

**Figure 1:**
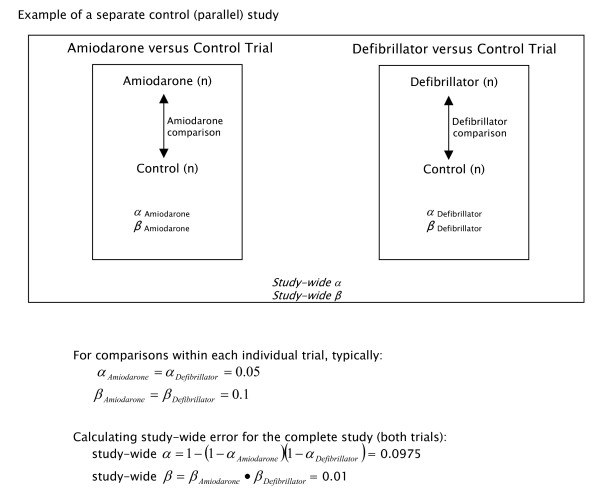
Depiction of a trial designed with 2 separate controls. The corresponding study-wide alpha (α) and beta (β) calculations are depicted below the design. The designated relative samples sizes are those for maximum efficiency. In the separate control trial design shown, two trials operate side-by-side, one comparing amiodarone to its own placebo control and the other comparing a defibrillator to its own (sham) control. The most efficient allocation of patients in this instance is 1:1.

**Figure 2:**
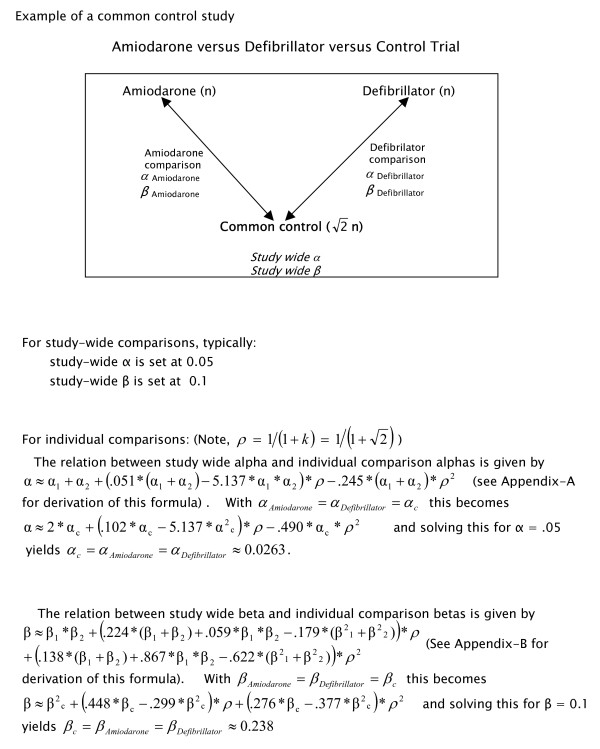
Depiction of a trial using a common control design testing the same clinical question as one with 2 separate controls (shown in figure 1). The corresponding study-wide alpha (α) and beta (β) calculations are depicted below the design. The designated relative samples sizes are those for maximum efficiency. In the common control design shown, the active treatments of amiodarone and a defibrillator are compared against a common control, which is administered an oral placebo. The most efficient allocation of patients to these treatments and the common control is in the ratio of 1:1:√2.

Figures 1 and 2 depict two studies that are identical in evaluating the same interventions, patient population, and having the same comparisons of interest. The separate control design (Figure 1) consists of two trials operating in parallel, one comparing, in this instance, amiodarone to its own placebo control, and the other comparing an implantable defibrillator to its own separate (sham) control. In contrast, the common control design (Figure 2) compares the same active treatments, amiodarone and a defibrillator, against a single common control group, which is administered an oral placebo. A prominent feature of this design, not encountered by the parallel two-group design, is the simple fact that variation in the control group affects all comparisons against it and hence induces correlations between findings. Indeed the pairwise correlation between comparison measures is ρ = 1/(1+k) if the common control group has k times as many patients as each comparison group (for example, the expected correlation ρ = 0.5 if the common control is the same size as each treatment group, that is, if k = 1).

### Allocation of patients to treatment and control groups

How patients are distributed (allocated) to intervention groups in common and separate control studies is based on a number of considerations. These include attempting to minimize costs from the various treatments to which they might be assigned, and maximizing statistical power. From the latter perspective, for a simple two-group comparison, where each intervention is compared against its own control, a 1:1 allocation of patients affords the greatest efficiency, that is, it minimizes the number of patients required for study. This is not the case for comparisons involving a common control. Specifically, if a fixed number of patients are allocated in a ratio of 1:1:...:1:k to a variety of m interventions (A_1_, A_2_,... A_m_), and a common control group, the efficiency is maximized when $\text{k}=\sqrt{\text{m}}$[6]. Thus in the instance where two treatment groups are compared to a common control (the scenario we consider in the remainder of this paper), the most efficient allocation of patients will be when the ratio of patients assigned to the control group is $\sqrt{2}$, resulting in an allocation ratio of 1:1:1.4.

### Defining and assigning study-wide error

In comparing common and separate control designs, one must distinguish study-wide error from the error assigned to individual comparisons. For either design, the study-wide alpha (α) is defined as the likelihood of seeing a benefit from either of the treatments (amiodarone or a defibrillator, for example), when no such benefit actually exists. Conversely, the study-wide beta (β) is defined as the likelihood of missing all benefits from the treatment interventions when both actually have benefit.

For a separate control trial design (Figure 1), one typically begins by assigning errors α_1 _and α_2 _for each comparison before calculating study-wide error. The study-wide α is then given by α = 1 - (1 - α_1_)(1 - α_2_). The study-wide β is given by β = β_1_·β_2_, where β_1 _and β_2 _are the betas assigned for each comparison [7]. If, for example, an α of 0.05 and a β of 0.1 were assigned to each comparison depicted in Figure 1, the study-wide α is 1 - (1 - .09)^2 ^or 0.0975 and the study-wide β is 0.1·0.1 or 0.01.

Conversely, in the common control design (Figure 2), one typically begins by assigning study-wide error (in this example α = 0.05 and β = 0.1) before calculating the values to be assigned for each individual comparison. In this instance, assuming that α and β are split evenly between two individual comparisons, then α_1_= α_2 _= approximately 0.0263 (from the formula derived in the Appendix (see additional file 1, section A)), and β_1 _= β_2 _= approximately 0.238 (see the formula derived in the Appendix (see additional file 1, section B)). Thus, even though designed to address the identical question, the typical manner of assigning error results in vastly different study-wide α and β for the common and separate control designs.

### Optimizing study efficiency

**Figure 3:**
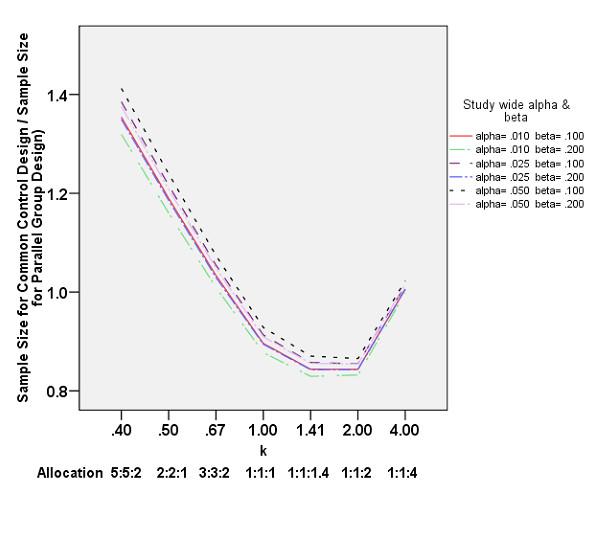
Efficiency of comparing two interventions, splitting the study wide alpha and beta equally, using the common control design compared to the optimal parallel group design (1:1 allocation) as a function of the allocation ratio 1:1:k across a wide spectrum of Type I (alpha) and Type II (beta) errors.

Figure 3 depicts the ratio of sample sizes required for a hypothetical intervention using common to separate control designs with all other design parameters being the same and with α_1 _= α_2 _and β_1 _= β_2_. When the proportion of subjects allocated to the common control group (k) is 0.75 < k < 2.5, the common control design is more efficient (resulting in approximately a 15% reduction in sample size for √2 ≤ k < 2); whereas the common control design is less efficient than the separate control design when k < 0.67 or k > 4.

### Subgroup analyses in a common control study

Variation in the common control will affect all comparisons made against it. This can lead to observing similar effects from differing treatments in a subgroup of patients, which may instill greater confidence in the presence of a subgroup effect, when, in fact, there should be less. The risk of variation in a group is proportional to the reciprocal of the square root of the group size. Thus the relative impact of the common control group on spurious subgroup findings increases as the allocation ratio decreases (for example, 1:1:1 rather than 1:1:$\sqrt{2}$). Notably, the presence of a common control may actually help determine whether a subgroup finding is related to the treatment under study, since, if due to the control group, all comparisons should be impacted, whereas if due to an intervention, only the comparison of that intervention against the control should be impacted.

### Calculating correlations

The magnitude of the expected correlation of comparisons between subgroups and a common control can be calculated. Presuming that outcomes in each subgroup are similar to the overall outcomes of the study, the expected correlation between the pairs of log relative risks for the comparisons of each intervention to the common control among subgroups is derived in the Appendix (see additional file 1, section C). The expected correlation is related to the reciprocal of the allocation ratio and (in the case where this is 1:1:1) is approximately equal to $\frac{1}{\sqrt{\left({\text{OR}}_{\text{1}}+\text{1}\right)\left({\text{OR}}_{\text{2}}+\text{1}\right)}}$ where OR represents the odds ratio for the overall effect of each treatment versus the common control.

## Results

**Figure 4:**
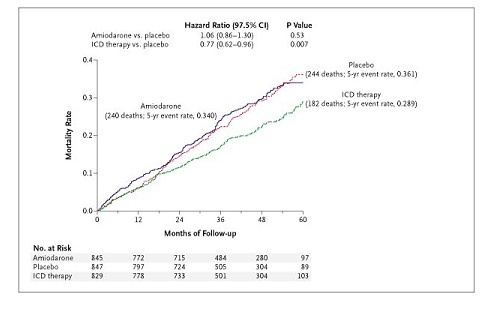
Primary outcome in Sudden Cardiac Death in Heart Failure Trial (SCD-HeFT). Kaplan-Meier estimates of death from any cause, comparing amiodarone and defibrillator therapy against their common control (placebo) group. (Copyright 2005, Massachusetts Medical Society [5]. All rights reserved.).

In SCD-HeFT, patients with NYHA Class II or III CHF, an ejection fraction ≤ 35%, and receiving standard heart failure therapies were randomly assigned to oral amiodarone, a defibrillator, or oral placebo (common control group) in a 1:1:1 ratio [5]. SCD-HeFT was powered at 90% to detect a 25% reduction in mortality at an alpha level of 0.025. The salient findings of the trial were that a defibrillator reduced total mortality by 23%, whereas amiodarone had no significant effect upon survival (Figure 4). Among the prespecified subgroup defined by CHF class, treatment with a defibrillator resulted in an appreciable reduction in mortality among patients with NYHA Class II CHF The respective hazard ratios for defibrillator therapy versus placebo (common control) and for amiodarone versus placebo (common control) were 0.54 (p = 0.001) and 0.85 (p = 0.17). Unexpectedly, no benefit of the defibrillator was apparent in those with NYHA Class III CHF, but that cohort was apparently harmed by amiodarone therapy. In class III CHF patients, the corresponding hazard ratios were 1.16 (p = 0.30) and 1.44 (p = 0.01). The reported p values for the tests of interaction between defibrillator therapy and NYHA class and between amiodarone therapy and NYHA class were 0.001 and 0.004, respectively.

### Clinical interpretation

That patients with NYHA CHF Class III in SCD-HeFT had no mortality benefit attributable to a defibrillator has been taken by some to suggest that prophylactic implantation of a defibrillator is not helpful for improving survival among patients in more advanced stages of CHF. Compared to patients with better ventricular function, it is argued that the prospect of death from worsening circulatory (pump) failure is proportionately higher in those with poor function and not something one would necessarily expect to be altered by a device that only affords treatment directed against arrhythmias [8]. This contrasts with results from other studies that indicated that a defibrillator improved outcome in patients with more advanced heart failure [1-4,9].

### Statistical insights

Although there are plausible clinical reasons for the different outcome in SCD-HeFT, its use of a common control design permits investigation of the possibility that the explanation lies in variation in the common control. Because of the relatively small proportion of patients allocated to the control limb, the trial was subject to a relatively large impact from the common control in subgroup analyses. Based on the 5 year mortality rate estimates provided in SCD-HeFT, the odds ratios for the effect of treatment with a defibrillator or amiodarone versus the common control were 0.719 and 0.912, respectively. This results in an expected correlation of the log of the relative risks for the comparisons (intervention versus common control) in the 7 subgroups from SCD-HeFT shown in Figure 4A without an *a priori *expectation of an interaction (that is, excluding the beta-blocker subgroup for whom an interaction with amiodarone might be expected) of $1/\sqrt{1.719•1.912}\approx 0.55$ (see formula in the Calculating Correlations section above). However, the observed correlation was 0.92. Assuming independence of the subgroups (an assumption supported by other studies having a similar population, such as the Dual Chamber and VVI Implantable Defibrillator (DAVID) trial, in which pairwise correlations between subgroups ranged from 0 to 0.13) [10], the probability of observing this magnitude of correlation (r = 0.92) in subgroup findings when the expected value should be 0.55 is p = 0.052 (see Appendix (additional file 1, section D)) [11].

**Figure 5:**
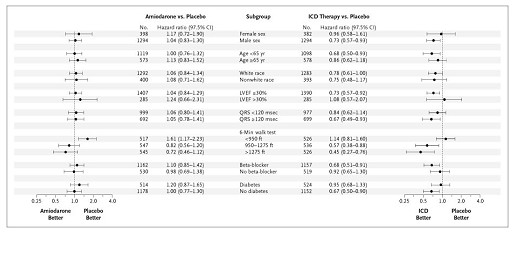
Hazard ratios for subgroups of patients treated with amiodarone or a defibrillator as compared with placebo in the Sudden Cardiac Death in Heart Failure Trial (SCD-HeFT). Abbreviations: ft = feet; ICD = implantable defibrillator; LVEF = left ventricular ejection fraction. (Copyright 2005, Massachusetts Medical Society [5]. All rights reserved.).

**Figure 6:**
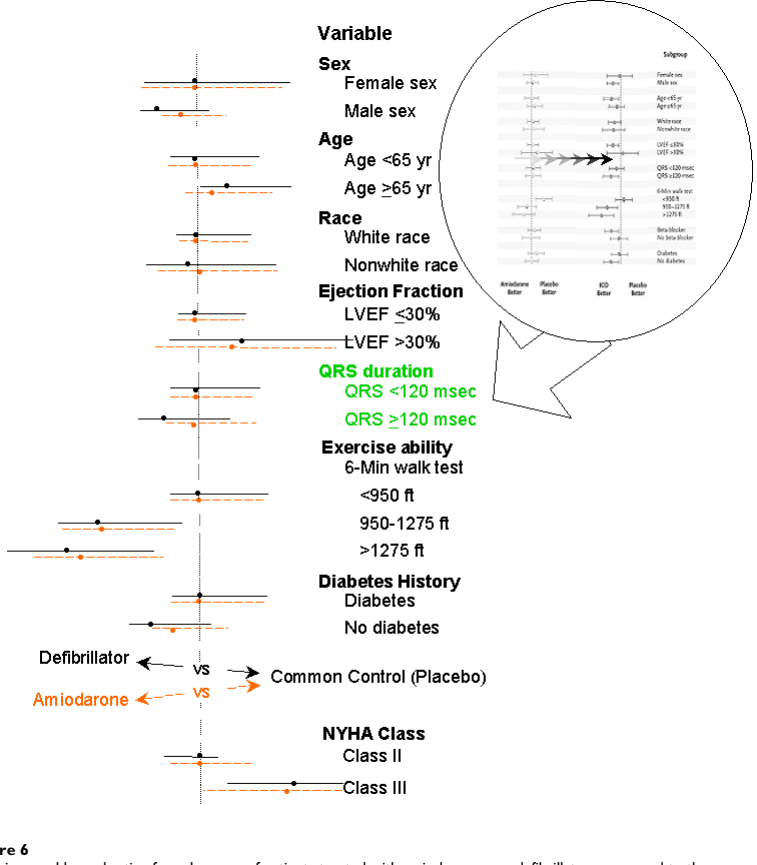
Superimposed hazard ratios for subgroups of patients treated with amiodarone or a defibrillator, compared to the common control in SCD-HeFT. The figure was created by superimposing the hazard ratios with confidence intervals reported in SCD-HeFT from the left portion of Figure 5 (depicting amiodarone versus placebo) upon those on the right (defibrillator therapy versus placebo) as shown in the circular inset (see text for specific construction). Dots with solid (or dashed) horizontal lines represent the hazard ratio and confidence intervals for the effect of treatment with a defibrillator (or amiodarone) versus the common control on outcome. The vertical dotted line represents the alignment of the hazard ratios for the topmost subgroup defined by each variable. Subgroups with discordant treatment effects are highlighted as green text. Abbreviations: ft = feet; LVEF = left ventricular ejection fraction. (Copyright 2005, Massachusetts Medical Society [5]. All rights reserved. Adapted with permission.).

This unexpected uniformity for subgroup responses between patients treated with amiodarone and those treated with a defibrillator is illustrated in Figures 5 and 6. Figure 5 shows the reported hazard ratios for subgroups of patients treated with amiodarone or a defibrillator as compared with placebo in SCD-HeFT. These were used to generate Figure 6. In depicting the superimposed hazard ratios shown in Figure 6, the absolute hazard ratio at the bottom of Figure 5 was ignored, and the hazard ratios were exactly aligned for the topmost subgroup defined by each variable (see insert in figure 6). For example, for the female and male subgroups that were defined by the sex variable, we exactly aligned the hazard ratios for the effect of treatment with amiodarone and treatment with a defibrillator in females. When the hazard ratios are "standardized" in this manner for one subgroup (e.g. females), the hazard ratios for the complementary subgroup(s) (e.g. males) indicate the quality and quantity of interaction between the treatments and the subgroups defined by that variable on outcome. This process was applied to each of the subgroups defined by the variables shown in Figure 5, excepting use of beta blockers (which might be expected to interact with treatment), and the NYHA subgroups were added at the bottom of the figure. The hazard ratios for the effect of treatment with amiodarone and treatment with a defibrillator on outcome are readily seen to be qualitatively, if not quantitatively, similar in virtually all subgroups. To find such a strong similarity in subgroup effects from two interventions whose effects proved to be far different in the main trial is surprising, and suggests that many of the subgroup findings resulted from what each of the treatments was compared against, that is, the common control.

### SCD-HeFT paradox

Among its individual subgroup comparisons, SCD-HeFT reported a significant benefit for defibrillator therapy in Class II CHF patients, a significant harm for amiodarone therapy in Class III CHF patients, and associated significant tests of interaction between each treatment and the NYHA class. However, the quantitatively similar effects seen among the NYHA subgroups in the bottom of figure 6 suggest that these interactions were likely the result of a common control phenomenon, not treatment. Indeed, if one eliminates the common control, the relative hazards for amiodarone versus defibrillator therapy (1.57 and 1.24 for Class II and Class III CHF respectively) were not significantly different (p~0.22). Thus the significant interactions reported by SCD-HeFT appear to depend upon the common control, and suggests that the failure to observe a benefit from an implanted defibrillator in patients with NYHA Class III heart failure was not a consequence of treatment, but more likely was caused by a variation in the common control group.

## Discussion

### Benefits of a common control

Trials using common rather than separate control groups have many desirable features. Perhaps the most important of these is conserving patients, the most valuable resource in a trial. A common control design with the optimal proportion of subjects allocated between treatment groups and the control can significantly reduce the number of patients required for study. From this perspective, by having a common control design with an allocation ratio of 1:1:1, less than the optimal of 1:1:√2, SCD-HeFT missed the opportunity to utilize its design to maximum efficiency. As a result, it may have enrolled more patients than required to test their hypotheses. It should be noted, however, that even with less than optimal allocation, the SCD-HeFT design was still more efficient than a parallel group design (figure 3).

In addition to these practical considerations, failure to take full advantage of study design efficiency also has ethical ramifications. The need to enroll fewer subjects in a trial means fewer persons being placed at risk for the interventions being tested. Because the optimal subject allocation ratio in a common control trial (1:1:√2 for a 2-treatment study) results in a higher proportion of subjects in the control group, this also prevents placing more patients in harm's way than necessary, were the trial's interventions to be proven harmful.

### Peril of a common control

Another potentially advantageous, but also perilous, feature of the common control design is the dependency such a design induces between comparisons of the active treatments with the common control. This design provides a useful tool to help evaluate the likelihood that a subgroup finding is actually due to a treatment effect. For example, seeing a positive subgroup finding for one treatment, but not for others, lends support to the presence of a true effect from treatment. Conversely, a positive subgroup finding for all the tested treatments is more likely to result from what the comparisons have in common, namely the control group, and hence supports their origin from chance variation in the common control rather than from the interventions themselves. In the latter instance, the peril lies in the natural tendency to develop greater confidence in such multiple positive findings as indicative of a real subgroup phenomenon, rather than their raising suspicion for the presence of a common control group effect. The controversial SCD-HeFT treatment paradox may represent a heretofore unrecognized example of this peril; puzzling until seen as a consequence of trial design, rather than a clinical phenomenon. This observation may explain and allay some of the concern for why the results for NYHA Class III CHF seen in SCD-HeFT did not agree with other studies.

### Limitations

Our analysis of SCD-HeFT was based on published descriptions of their design and findings. We did not have access to the patient specific data and hence could not examine the relationships between various subgroups in more detail. Furthermore, our statistical arguments are intended to provide further insight and guidance in interpretation, not necessarily exclude other plausible explanations for the results of these trials.

## Conclusion

Common control trials compare several intervention groups to a single rather than to separate control groups. Such trials can be more efficient than those using separate control groups, but induce a dependence between the comparisons of their treatments with the common control. The efficiency of a common control trial's design is reduced and even negated if the allocation ratio between treatment and control groups strays far from the optimal, and its risk for fortuitous subgroup findings will be enhanced when the allocation to the common control is smaller than optimal. These principles may explain some of the paradoxical and discordant findings in recent trials, and can serve to guide the design and analyses of future clinical trials using a common control.

## List of abbreviations

CHF: Congestive heart failure; NYHA: New York Heart Association; SCD-HeFT: Sudden Cardiac Death in Heart Failure trial.

## Competing interests

The authors declare that they have no competing interests.

## Authors' contributions

PJK and APH both conceived of the study and participated in its design and presentation of results. AHP performed the statistical analyses. All authors read and approved the final manuscript.

## Supplementary Material

Additional file 1 Appendix. The appendix is provided as a reference for the reader with interest in how some of the calculations under discussion were derived, or for use in the design of future trials. It is divided into sections A-D as referred to in the text. Click here for file
