# Supplementary material for: Pearls and perils of an implantable defibrillator trial using a common control: implications for the design of future studies
Source: Trials. 2008 May 2;9:24. doi: 10.1186/1745-6215-9-24 (PMC2397377; doi:10.1186/1745-6215-9-24)
Supplement: Additional file 1 — Appendix. The appendix is provided as a reference for the reader with interest in how some of the calculations under discussion were derived, or for use in the design of future trials. It is divided into sections A-D as referred to in the text. [file 1745-6215-9-24-S1.doc]

Appendix

The appendix is provided as a reference for the reader with interest in how some of the calculations under discussion were derived, or for use in the design of future trials. Because the examples and applications are based on dichotomous outcomes, the derivations in the appendices are presented in those terms. However, the derivations depend only on the central limit theorem (that is, that the sample mean tends to a normal distribution), and hence the results are equally applicable to continuous as well as dichotomous outcomes.

Appendix

**A. Formulae for deriving the study-wide alpha for a common control trial:**

Let X=A-C, Y=B-C be bivariate normal N$\left(\left(0,0\right),1,1,\text{ρ}\right)$. Let Z_x_ and Z_y_ be critical values and $\text{α}_{\text{x}}=2\bar{\Phi}\left(\text{Z}_{\text{x}}\right)$ and $\text{α}_{\text{y}}=2\bar{\Phi}\left(\text{Z}_{\text{y}}\right)$ be the corresponding alpha levels, where $\Phi$ is the cumulative normal, $\bar{\Phi}=1-\Phi$, and $\phi$ is the normal density. Then the study wide $\text{α}\approx\text{α}_{\text{x}}+\text{α}_{\text{y}}+\left(.051*\left(\text{α}_{\text{x}}+\text{α}_{\text{y}}\right)-5.137*\text{α}_{\text{x}}*\text{α}_{\text{y}}\right)*\rho-.245*\left(\text{α}_{\text{x}}+\text{α}_{\text{y}}\right)*\rho^{2}$ (1) with an error less than 2.5% for $\text{.01 }<\text{α}<.1$.

*Proof:* The distribution of X,Y is given by

$$\text{f(x,y})=\frac{1}{2\text{π}\sqrt{1-\text{ρ}^{2}}}\text{e}^{-\frac{1}{2}\frac{1}{1-\text{ρ}^{2}}\left(\text{x}^{2}-2\text{ρxy}+\text{y}^{2}\right)}$$

$$=\frac{1}{2\text{π}\sqrt{1-\text{ρ}^{2}}}\text{e}^{-\frac{1}{2}\left(\frac{\text{x}-\text{ρy}}{\sqrt{1-\text{ρ}^{2}}}\right)^{2}}\bullet\frac{1}{\sqrt{2\text{π}}}\text{e}^{-\frac{1}{2}\text{y}^{2}}$$

α = Prob (Type I error) = Prob (**|**X**|**>Z_x_ and/or **|**Y**|**>Z_y_)

= Prob (**|**X**|**>Z_x_)+Prob(**|**Y**|**>Z_y_)-Prob(**|**X**|**>Z_x_ and **|**Y**|**)>Z_y_)

= Prob(X>Z_x_ and Y>Z_y_)-2Prob (X>Z_x_ and Y<-Z_y_)

Prob(X>Z_x_ and Y>Z_y_), since ρ>0.

Now Prob(X>Z_x_ and Y>Z_y_)

=$\int_{\text{Z}_{\text{y}}}^{\infty} \phi\left(\text{y}\right)\int_{\text{Z}_{\text{x}}}^{\infty} \phi_{\text{ρy},\sqrt{1-\text{ρ}^{2}}}\left(\text{x}\right)\text{dxdy}$= $=\frac{1}{2}\int_{0}^{\alpha_{y}} \Phi\left(\frac{\text{ρ}\Phi^{\text{-1}}\left(1-y/2\right)-\Phi^{\text{-1}}\left(1-\alpha_{x}/2\right)}{\sqrt{1-\text{ρ}^{2}}}\right)\text{dy}$, and the result is then obtained by integrating numerically over a range of alphas and rho and fitting the resulting data so the fitted values were within 2.5% of the data. *q.e.d.*

**B. Formulae for deriving the study-wide beta for a common control trial**:

Under the alternative $\left(\text{X,Y}\right)$~ N $\left(\left(\Delta,\Delta\right),1,1,\text{ρ}\right)$, then the study wide $\begin{matrix} \text{β}\approx\text{β}_{\text{x}}*\text{β}_{\text{y}}+\left(.224*(\text{β}_{\text{x}}+\text{β}_{\text{y}})+\text{.059*β}_{\text{x}}*\text{β}_{\text{y}}-.179*(\text{β}^{2}_{\text{x}}+\text{β}^{2}_{\text{y}})\right)\text{*ρ} \\ +\left(\text{.138*}(\text{β}_{\text{x}}+\text{β}_{\text{y}})+\text{.867*β}_{\text{x}}*\text{β}_{\text{y}}-.622*(\text{β}^{2}_{\text{x}}+\text{β}^{2}_{\text{y}})\right)*\text{ρ}^{2} \end{matrix}$ (2)

with an error less than 2.5% for $\text{.1 }<\beta<.4$.

*Proof:*

$\text{β}$=Prob(X<Z_x_ and Y<Z_y_|Δ). Now $\text{f}_{\Delta}\left(\text{x,y}\right)=\frac{1}{2\text{π}\sqrt{1-\text{ρ}^{2}}}\text{e}^{-\frac{1}{2}\frac{1}{1-\text{ρ}^{2}}\left[\left(\text{x}-\Delta\right)^{2}-2\text{ρ}\left(\text{x}-\Delta\right)\left(\text{y}-\Delta\right)+\left(\text{y}-\Delta\right)^{2}\right]}$

=$\frac{1}{\sqrt{2\text{π}}\sqrt{1-\text{ρ}^{2}}}\text{e}^{-\frac{1}{2}\left(\frac{\text{x}-\text{Δ}-\text{ρ}\left(\text{y}-\text{Δ}\right)}{\sqrt{1-\text{ρ}^{2}}}\right)^{2}}\bullet\frac{1}{\sqrt{2\text{π}}}\text{e}^{-\frac{1}{2}\left(\text{y}-\text{Δ}\right)^{2}}$ so

$\text{β}$=$\int_{-\infty}^{\text{Z}_{\text{y}}} \phi\left(\text{y}-\text{Δ}\right)\int_{-\infty}^{\text{Z}_{\text{x}}} \phi_{\text{Δ}+\text{ρ}\left(\text{y}-\text{Δ}\right)\text{,}\sqrt{1-\text{ρ}^{2}}}\left(\text{x}\right)\text{dxdy}$

=$\int_{-\infty}^{\text{Z}_{\text{y}}} \phi\left(\text{y}-\Delta\right)\Phi\left(\frac{\text{Z}_{\text{x}}-\Delta-\text{ρ}\left(\text{y}-\Delta\right)}{\sqrt{1-\text{ρ}^{2}}}\right)\text{dy}$ (let $\text{y}=\text{y}-\Delta$)

=$\int_{-\infty}^{\text{Z}_{\text{y}}-\Delta} \phi\left(\text{y}\right)\Phi\left(\frac{\text{Z}_{\text{x}}-\text{Δ}-\text{ρy}}{\sqrt{1-\text{ρ}^{2}}}\right)\text{dy}$ $=\int_{0}^{\beta_{\text{y}}} \Phi\left(\frac{\Phi^{\text{-1}}\left(\beta_{x}\right)-\text{ρ}\Phi^{\text{-1}}\left(\text{y}\right)}{\sqrt{1-\text{ρ}^{2}}}\right)\text{dy}$ , and the result is then obtained by integrating numerically over a range of betas and rho and fitting the resulting data so the fitted values were within 2.5% of the data. *q.e.d.*

Application: Let the observations be a_i_, b_j_, c_k_ and set , . Then Var$\bar{\text{X}}$=Var$\bar{\text{Y}}=\frac{\text{Var}_{\text{a}}}{\text{N}}+\frac{\text{Var}_{\text{c}}}{\text{kN}}$. Set $\text{X}=\bar{\text{X}}\text{/}\sqrt{\text{Var}\bar{\text{X}}}$ and $\text{Y}=\bar{\text{Y}}\text{/}\sqrt{\text{Var }\bar{\text{Y}}}$. Then X and Y satisfy the criteria of the lemma. Note that if |E(a-c)|=$\delta$, $\Delta=\delta/\sqrt{\frac{\text{Var}_{\text{a}}}{\text{N}}+\frac{\text{Var}_{\text{c}}}{\text{kN}}}$. Thus solving (1) for $\alpha_{\text{x}}$=$\beta_{\text{y}}$ for a given study wide$\alpha$, and (3) for $\text{β}_{\text{x}}$=$\text{β}_{\text{y}}$ for a given study wide$\text{β}$, the sample size, N(2+k), is given by solving . E.g., if $\text{θ}_{\text{T}}$ is the expected rate under treatment and $\text{θ}_{\text{C}}$ the expected control rate, $\delta=\left|\text{θ}_{\text{T}}-\left.\text{θ}_{\text{C}}\right|\right.$ and $\Delta=\delta/\sqrt{\frac{\text{θ}_{\text{T}}\left(1-\text{θ}_{\text{T}}\right)}{\text{N}}+\frac{\text{θ}_{\text{C}}\left(1-\text{θ}_{\text{C}}\right)}{\text{kN}}}$.

C. Formulae deriving the expected correlation (log of the relative risks) between the comparison of each intervention to the common control, in a given subgroup. These calculations are derived under the subgroup null, that is, presuming outcomes in each subgroup are similar to the overall outcomes.

Consider the case of a binary outcome and suppose the overall rates are $\mu_{\text{C}}$, $\mu_{1}$and $\mu_{2}$. Suppose baseline criteria identify subgroups (by the 1:1:k allocation) of size n, n and kn. Often subgroups are chosen because of a presumed greater or lesser underlying risk. Thus, under the subgroup null, the rates in the subgroups can be represented by $\text{θ}_{\text{C}}=\lambda\mu_{\text{C}}$, $\text{θ}_{1}=\lambda\mu_{\text{C}}$, and $\text{θ}_{2}=\lambda\mu_{\text{C}}$. Then, under the subgroup null, $\tilde{\text{θ}}_{\text{C}}$~N$\left(\text{θ}_{\text{C}},\frac{\text{θ}_{\text{C}}\left(1-\text{θ}_{\text{C}}\right)}{\text{kn}}\right)$, $\tilde{\text{θ}}_{1}$~N$\left(\text{θ}_{1},\frac{\text{θ}_{1}\left(1-\text{θ}_{1}\right)}{\text{n}}\right)$ and similarly for $\tilde{\text{θ}}_{2}$, and the relative risks are S_1_=$\frac{\text{θ}_{1}}{\text{θ}_{\text{C}}}=\frac{\mu_{1}}{\mu_{\text{C}}}$ and S_2_=$\frac{\text{θ}_{2}}{\text{θ}_{\text{C}}}=\frac{\mu_{2}}{\mu_{\text{C}}}$. Let X=log($\tilde{\text{S}}_{1}$)=log($\tilde{\text{θ}}_{1}$)-log($\tilde{\text{θ}}_{2}$) and Y=log($\tilde{\text{S}}_{2}$). To a first order of approximation, if W~$\left(\text{μ,σ}^{2}\right)$, log(W)~(log ($\text{μ}$),$\frac{\text{σ}^{2}}{\text{μ}^{2}}$) so, under the null X~ N(log ($\mu_{1}$)-log($\mu_{\text{C}}$),$\frac{\frac{1-\text{θ}_{1}}{\text{θ}_{1}}+\frac{1-\text{θ}_{\text{C}}}{\text{kθ}_{\text{C}}}}{\text{n}}$) and similarly for Y.

Then under the subgroup null, corr(X,Y)=$\frac{\frac{1-\text{θ}_{\text{C}}}{\text{kθ}_{\text{C}}}}{\sqrt{\left(\frac{1-\text{θ}_{1}}{\text{θ}_{1}}+\frac{1-\text{θ}_{\text{C}}}{\text{kθ}_{\text{C}}}\right)\left(\frac{1-\text{θ}_{2}}{\text{θ}_{2}}+\frac{1-\text{θ}_{\text{C}}}{\text{kθ}_{\text{C}}}\right)}}$ $=\frac{1}{\sqrt{\left(\text{kOR}_{1}+1\right)\left(\text{kOR}_{2}+1\right)}}$ where OR is the odds ratio for the effect of treatment versus the common control in the subgroups. Thus the corr(X,Y) decreases approximately linearly from 1 to 0 as k increases from 0. Let $\text{R}=\text{corr(X,Y)}$when $\lambda=1$(that is when the risk in the subgroup is the same as the overall risk), and write

$\Delta=1-1/\lambda$. Then, as a function of $\Delta$, under the null, $\text{corr(X,Y)(}\Delta\text{)}\approx\text{R}+\text{T}\Delta$, where R and T can be estimated from the entire sample. Assuming then that the set of subgroups evaluated has risks centrally distributed about $\lambda=1$, the expected correlation of the pairs of log relative risks across the subgroups would be R.

**D. Calculating the probability for the magnitude of correlation observed between treatment subgroups in common control trials (exemplified for SCD-HeFT):**

The transformation W=$\frac{1}{2}$(log_e_(1+r)-log_e_(1-r)) has variance $\text{σ}_{\text{w}}^{2}=\frac{1}{\text{n}-3}$ [11]. Using this transformation, the probability of observing the magnitude of correlation (r= 0.92) as was seen in SCD-HeFT, when the expected value should have been 0.55, was $\bar{\Phi}(1.94)=0.052$.
